# Extraction Techniques and Modification Methods for Regulating the Structural and Functional Properties of Oleosome-Associated Proteins: A Review

**DOI:** 10.3390/foods14223849

**Published:** 2025-11-11

**Authors:** Yufan Sun, Mingming Zhong, Muhammad Safiullah Virk, Qin Liu, Qiufang Liang, Haile Ma, Xiaofeng Ren

**Affiliations:** 1 School of Food and Biological Engineering, Jiangsu University, 301 Xuefu Road, Zhenjiang 212013, China; yufan.sun@ujs.edu.cn (Y.S.); mingming.zhong@ujs.edu.cn (M.Z.); safiullahvirk@hotmail.com (M.S.V.); liuqin496043732@163.com (Q.L.); lqf@ujs.edu.cn (Q.L.); mhl@ujs.edu.cn (H.M.); 2 Institute of Food Physical Processing, Jiangsu University, 301 Xuefu Road, Zhenjiang 212013, China

**Keywords:** oleosome-associated proteins, protein extraction, protein modification, structure, functional properties

## Abstract

In recent years, oleosome-associated proteins (OPs) have gained increasing attention in the food and nutrition sectors due to their balanced amino acid composition and excellent functional properties. However, their low extraction yield, high hydrophobicity, and poor solubility hinder broader application in food systems. This review provides a concise overview of OPs’ structural features, current extraction strategies, and the impact of modification techniques on their structural and functional attributes. Special emphasis is placed on hybrid extraction methods that integrate physical treatments (e.g., ultrasound, heating, colloid milling) with traditional chemical approaches to enhance yield while preserving protein functionality. Furthermore, the review discusses how physical and chemical modifications effectively regulate OPs’ solubility, emulsifying capacity, aggregation behavior, and self-assembly characteristics. The regulatory mechanisms of different processing conditions on protein conformation and intermolecular interactions are summarized to guide functional optimization. Finally, the current technical challenges are outlined and future research directions are proposed, including the industrial scaling of hybrid extraction, precise control of structural modification, and application of OPs in emulsified and gel-based delivery systems. This work offers theoretical insight and practical guidance for the high-value utilization of OPs in food and related industries.

## 1. Introduction

**Figure 1:**
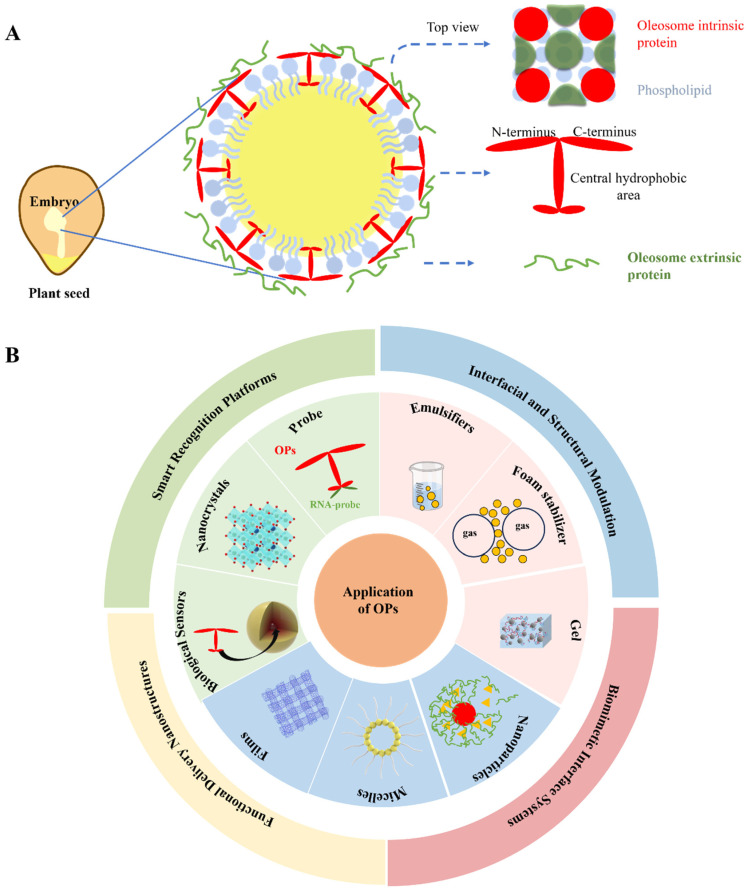
(**A**) Diagram of oleosome structure; top view of oleosome oil–water interface (phospholipids and oleosome proteins not drawn to scale), (**B**) Applications of OPs.

Oleosome-associated proteins (OPs) are key components of oleosomes (oil bodies), the organelles responsible for storing oils in plant cells, and play a critical role in lipid metabolism and accumulation [1]. As essential stabilizing elements of oleosomes, OPs are embedded on their surface, with their hydrophobic core anchored in the triglyceride matrix and hydrophilic regions spanning the oil–water interface, as illustrated in Figure 1A [2,3]. As naturally occurring interfacial-stabilizing proteins, OPs account for less than 2% of the total composition within oleosomes (specifically referring to endogenous proteins), yet they are capable of stabilizing over 70% of the oil phase, demonstrating remarkable potential as super emulsifiers [4,5]. Compared with conventional plant proteins, which lack inherent interfacial activity, OPs exhibit significant advantages in emulsification capacity, physicochemical stability, foaming ability, and gelling properties [6]. Mostly, OPs are widely employed as functional additives and food structure modifiers in the food industry. In addition, due to the abundance of both hydrophobic and polar amino acids, OPs possess structural adaptability, enabling them to adjust their conformation in response to environmental changes and thus stabilize complex food structures. Their small molecular size further facilitates their compatibility with various complex food systems [7]. OPs can self-assemble or co-assemble with other biopolymers into carrier systems with diverse scales and functional properties, including microparticles [8], nanopolymers [9], nanocrystals [10], micelles [11], and RNA probes [12] (as illustrated in Figure 1B). As such, OPs have emerged as key functional agents in food and biosciences, with broad application prospects in functional food systems and biopharmaceuticals.

However, OPs as components of plant oleosomes, face certain limitations in practical processing, similar to most plant proteins. For instance, OPs possess high surface hydrophobicity, which makes them prone to aggregation in solution [13]. Additionally, their poor solubility at neutral pH and low extraction yield limits their application in the food industry [14]. Furthermore, the digestibility of OPs presents a critical challenge. During extraction, the presence of antinutritional factors and lipids forms complex protein-lipid structures with OPs, hindering the action of digestive enzymes. Protein crosslinking and aggregation further impede effective breakdown by digestive fluids [15]. Thus, modifying OPs is an essential factor to amend their functional and physicochemical properties. Modification methods must prioritize safety and maintain nutritional value, with common approaches including thermal treatment, ultrasonic processing, pH adjustment, and surfactant modification [16]. However, while these conventional modification methods enhance certain properties, they often compromise the native molecular structure and key nutritional components of OPs, reducing their activity and bioavailability, and thereby limiting their practical applications.

In comparison, selecting appropriate extraction techniques preserves OPs’ functional integrity along with enhancing their yield significantly, making it a key solution to most challenges. Protein extraction is a foundational step in food science and technology research, with its efficiency and effectiveness directly impacting protein quality and application potential [17]. This process involves a variety of traditional and innovative techniques. Traditional methods (chemical extraction methods) have become the industry standard due to their wide application. However, these methods are limited by extraction time, solvent, pH value, and temperature, probably leading to lower extraction efficiency and loss of functional integrity [18]. For this reason, researchers’ attention is escalating to innovative hybrid extraction technologies, such as chemical and surfactant combined extraction, physical and chemical combined extraction, and ultrasound-assisted extraction technologies. These advanced techniques, especially working in combination, significantly improve protein yield and minimize degradation, thereby optimizing the efficiency and effectiveness of the extraction process. Thus, OPs’ optimized and enhanced functionality critically depends on the selection of appropriate extraction techniques and modification methods, ultimately influencing the scope and depth of OPs’ application in the food industry.

Previous studies have primarily focused on the composition, structure, and extraction methods of oleosomes derived from various plant sources [3,19]. However, there is currently a lack of systematic research and in-depth discussion regarding the specific effects of different extraction techniques on the structural and functional properties of OPs. Most existing reviews emphasize the overall physicochemical properties of oleosomes, while detailed investigations into how different extraction methods modulate the structural stability and functional characteristics of OPs themselves remain limited. Therefore, this review provides a comprehensive analysis of current extraction and modification techniques for OPs and offers guidance for future research. While the application of OPs is still at the experimental stage, their structural and functional versatility highlights promising prospects for future use in food and pharmaceutical development.

## 2. Current Research Status

**Figure 2:**
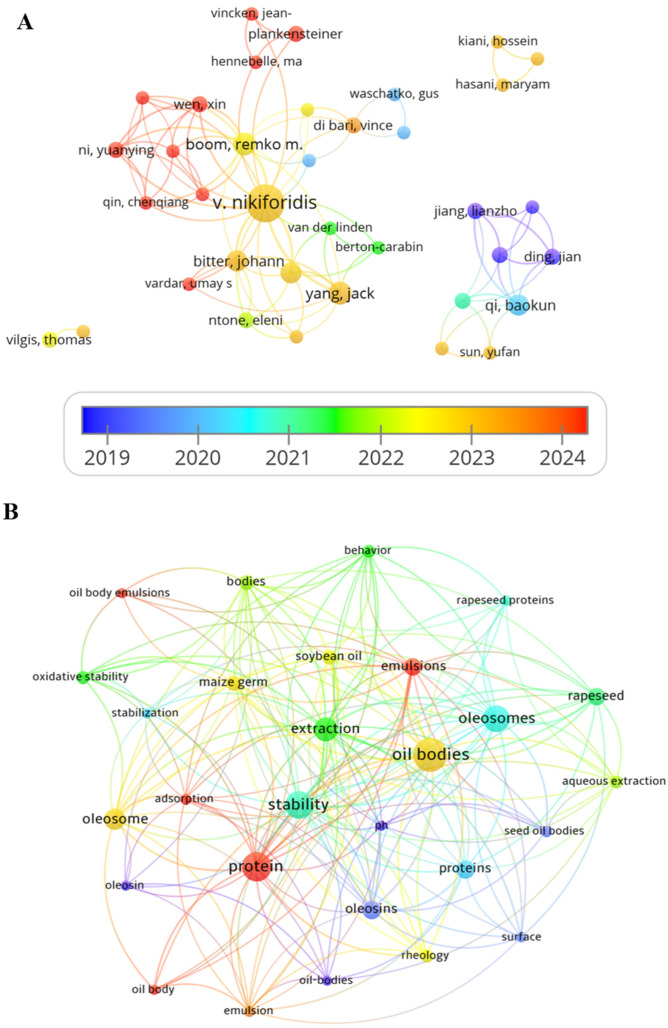
(**A**) Author Cooperation Map and Co-Cooperating Authors-Based Clusters (2019–2024); (**B**) Knowledge mapping of keyword co-occurrence network based on the VOSviewer in OPs.

OPs are unique to plant oleosomes and play a decisive role in maintaining the stability and integrity of oleosome structures. In recent years, increasing attention has been paid to the extraction and processing of OPs. To investigate the current research status in this field, a bibliometric analysis was conducted using data retrieved from the Web of Science Core Collection. Publications between 1 January 2019 and 31 December 2024, containing the terms “oleosome,” “oil body,” and “oleosome-associated proteins (OPs)”, were collected. The dataset included journal articles, proceedings papers, and book chapters, while records with a final publication year of 2025 were excluded to ensure dataset completeness and reproducibility. The analyses were performed using R software (version 4.3.11, Bibliometrix package), Microsoft Excel 2019, VOSviewer (version 1.6.18), and CiteSpace (version 6.2.R4). Specifically, Bibliometrix and VOSviewer were used to construct author and institutional collaboration networks and analyze co-citation relationships, while citeSpace was applied to generate keyword burst and cluster visualization maps (Figure 2A). The analysis revealed that Nikiforidis, Remko, Qi, Leonard and Johannes were the top five most productive authors. Among them, Nikiforidis and Leonard are recognized as core researchers, mainly focusing on the interfacial structure and stabilization mechanisms of oleosomes. Johannes Bitter has contributed significantly to the development of extraction processes and the investigation of functional properties of oleosome proteins. Qi et al.’s research primarily concentrates on the structural features of oleosomes and their responses to processing and environmental factors, although his collaboration with other mainstream research groups remains limited. Meanwhile, Wen and Ni have recently conducted collaborative studies with Remko’s group, focusing on the optimization of OPs extraction and functional modification. These findings indicate that a relatively mature research network has formed in this field, although cooperation among some researchers could still be strengthened.

Analysis of keywords related to oleosomes (Figure 2B) from 2019 to 2020 frequently highlighted terms such as “stability” and “pH,” reflecting a focus on emulsion stability under processing, environmental, and storage conditions. Since 2021, however, the research focus has gradually shifted to the interfacial structure of oleosomes, particularly emphasizing the relationship between interfacial properties and keywords such as “rheological properties”, “interfacial proteins” and “oleosin” (the major protein component of OPs). Notably, the composition and structural changes of oleosome proteins and their correlation with emulsion functionality have remained central themes. Moreover, various extraction techniques have been widely applied to explore structural and functional changes in oleosomes, aiming to improve extraction yield, oxidative stability, gelling behavior, and emulsification properties, thereby broadening practical applications. Since 2023, the effects of different extraction methods on oleosome structure and functional performance have become a research hotspot. However, there is still a lack of systematic and in-depth studies on the mechanisms by which different extraction methods influence the structural and functional properties of OPs, which is the core focus of this review. In addition, current research on oleosomes and OPs covers a wide range of plant sources, including major oilseed crops such as soybean, sesame, and rapeseed, as well as other edible or specialty plants like quinoa, hemp, and coconut. Although oleosomes from different plant sources share certain structural and functional similarities, they also exhibit distinct differences, which provides a diversified foundation for the study and application of OPs.

## 3. Structure, Composition, and Key Sources of OPs from Various Sources

### 3.1. Structural Characteristics of Intrinsic Proteins: Oleosin, Caleosin, and Steroleosin

**Figure 3:**
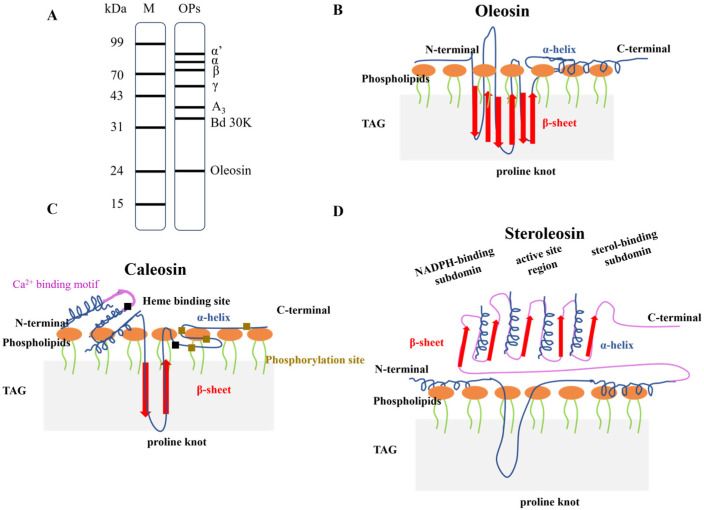
Diagram of the intrinsic protein secondary structure of OPs: (**A**) Oleosin, (**B**) Caleosin, (**C**) Steroleosin (**D**) SDS-PAGE analysis of soybean OPs.

OPs are unique proteins found in plant oleosomes and play a decisive role in maintaining the structural stability and integrity of oleoosmes. As noted by Huang [20,21], OPs form a dense and stable protective layer with phospholipids at the oleosome interface, effectively preventing droplet coalescence through steric hindrance and electrostatic repulsion. OPs primarily consist of three intrinsic proteins: oleosin, caleosin, and steroleosin (as shown in Figure 3A). Each of these proteins possesses distinct structural characteristics and functionalities, collectively contributing to the stability and functionality of oleosomes.

Among them, oleosin is the most abundant structural protein in OPs, with a molecular weight of 15–26 kDa [22]. Its central hydrophobic peptide chain is embedded in the lipid core of the oleosome, while the hydrophilic N- and C-termini are exposed on the oleosome surface (as shown in Figure 3B), forming steric hindrance and electrostatic repulsion that effectively prevent droplet aggregation and coalescence [23]. In addition, Hu et al. reported that oleosin plays an important regulatory role in oleosome particle size and seed development in plants [24]. In addition to oleosin, another protein, caleosin, which is larger than oleosin (27 to 29 kDa) has also been isolated and identified [25]. Its structure is similar to that of oleosin, consisting of three structural domains, but features a shorter central hydrophobic segment and a longer hydrophilic region containing a Ca^2+^-binding EF-hand motif, enabling it to more effectively stabilize the oleosome structure (as shown in Figure 3C) [26]. Consequently, oleosomes coated with caleosin are more stable than those coated only with oleosin [25,27]. Steroleosin is the least abundant but the largest intrinsic oleosome protein, with a molecular weight of 39–41 kDa [28]. Its N-terminus is hydrophobic, similar in length to that of caleosin, and anchors to the oleosome membrane. The C-terminus contains binding sites for NADPH and sterol dehydrogenase, and extends into a hydrophilic domain homologous to sterol-binding dehydrogenase [29,30], with the structure shown in Figure 3D.

### 3.2. Interaction and Influence of Extrinsic Proteins on Oleosomes

In addition to the three intrinsic proteins mentioned above, the surface of oleosomes also adsorbs various extrinsic proteins, including plant storage proteins (7S globulin, 11S globulin, and 2S albumin) and active proteins (lipoxygenase, trypsin inhibitors, Bd k30, P34) [31] (as shown in Figure 3A). These extrinsic proteins are located in the plant seed proteome and do not participate in oleosome synthesis. However, during wet milling, a large number of extrinsic proteins and oleosomes are simultaneously released from seed cells. During the centrifugal enrichment process of oleosomes, the extrinsic proteins interact with the three oleosome intrinsic proteins through electrostatic interactions [32]. Ishii et al. [33] found that the interface thickness of crude oleosomes, which had not been purified, was 1.6 times that of the interface thickness of pure oleosomes. Therefore, extrinsic proteins can affect the physical and chemical properties of oleosomes, including increasing particle size, lowering the isoelectric point, and accelerating oxidation. Therefore, there is a need to extract and separate the intrinsic and extrinsic proteins from oleosomes and explore their roles and mechanisms within oleosomes, and to avoid its adverse effects on the functional properties and practical applications.

### 3.3. Variations in OPs Composition Among Different Plant Sources

To obtain OPs with different structures, scientists have continuously adjusted extraction and modification techniques, aiming to improve their functional properties. Existing studies show that through these techniques, extrinsic proteins in OPs can be selectively removed or retained based on specific application needs, thus optimizing the purity of OPs and enhancing their functional characteristics. This will further contribute to a deeper understanding of the mechanisms of OPs, advancing their potential applications in the food industry.

In addition to differences in protein types, oleosomes from different plant sources also exhibit significant variations in protein content and lipid composition, which directly determine the extraction potential and application value of oleosome-associated proteins (OPs). Soybean oleosomes have a relatively high OPs content, accounting for approximately 8.2% of the oleosome dry weight, with neutral lipids making up about 85.9% and phospholipids about 5.4%. The relatively balanced protein-to-lipid ratio makes soybean an ideal source material for OPs extraction [34]. Safflower oleosomes also have a high protein content (around 7.3%), but a higher proportion of neutral lipids (approximately 92.6%), indicating that additional purification steps may be required during OPs extraction to remove excess lipids [35]. In contrast, oleosomes from nut sources such as hazelnut and coconut have extremely low protein contents (2.48% and only 0.6%, respectively), with lipid contents of 83.07% and 81.1%, respectively. This suggests that oleosomes from nuts contain very limited extractable OPs and are not optimal raw materials for OPs functional studies or extraction process development. Furthermore, the oleosome protein contents of oil crops such as peanut, sesame, rapeseed, and camellia seed fall between those of nuts and soybeans (ranging from 0.8–5.1%), while their lipid contents all exceed 85% [36]. In practical applications, OPs extraction from these materials may require additional optimization of purification processes to improve purity and reduce lipid interference. Therefore, clarifying the OPs potential of different plant sources based on protein content and lipid ratio is crucial for effectively designing OPs extraction strategies and function-oriented studies, particularly in the separation, purification, and functional optimization of intrinsic proteins (such as oleosin, caleosin, and steroleosin) and extrinsic proteins (such as 7S and 11S globulins).

## 4. Modification of OPs Through Extraction Techniques

Choosing the appropriate OPs extraction method is crucial for maximizing their yield and determining structure and physical properties. Different extraction methods can significantly impact the functional properties of OPs, such as solubility, emulsifying ability, and interfacial characteristics, thereby greatly influencing their potential applications in the food industry [37]. Choosing different protein extraction methods depends on the intended final product and the available technical conditions. Traditional protein extraction methods are primarily classified into chemical and physical methods, while non-conventional extraction methods refer to hybrid techniques that combine traditional methods with other sophisticated technologies to enhance protein extraction efficiency and yield [38].

### 4.1. Traditional Extraction

**Figure 4:**
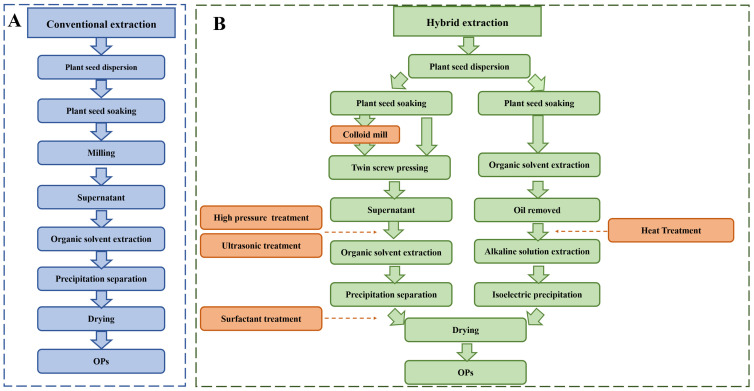
The process of OPs extraction: (**A**) conventional extraction; (**B**) hybrid extraction.

Since OPs are exclusively found in oleosomes and play a vital role in plant lipid metabolism, conventional physical methods (such as soaking, grinding, and sieving) are ineffective in removing the lipids associated with OPs. While enzyme-assisted extraction can improve protein extraction efficiency, the hydrolytic action of proteases may lead to the breakdown of oleosin, which is detrimental to OPs extraction [39]. Therefore, chemical extraction methods are preferred to isolate OPs. Chemical extraction effectively disrupts the lipid-protein association, improving extraction efficiency while ensuring protein purity. The chemical extraction methods for OPs are shown in Table 1. The traditional extraction process for OPs generally involves four steps, enrichment, extraction, precipitation, and drying, as illustrated in Figure 4.

#### 4.1.1. Enrichment of OPs Using Chemical Reagents

Typically, plant seeds are soaked in aqueous media for enrichment, followed by mixing or pressing to disrupt the cell wall and release the OPs. Common grinding media include urea, sucrose, deionized water, salt, alkaline solutions, and buffer solutions (such as Tris-HCl and PBS). Initially, Tzen [26] laid the foundation for OPs enrichment using a mixed buffer solution (sucrose, EDTA, KCl, MgCl_2_, DTT, and Tricine). However, as research advanced, the extraction medium was simplified, with the simplified media offering advantages in extraction efficiency and purity. Notably, using 9 M urea for OPs enrichment showed better purity and stability, as it was less affected by extrinsic proteins, though urea’s application in food-grade products remains limited. Nowadays, the solubility of protein can be solved by pH and salt so that OPs extraction efficiency is improved. Romero-Guzmán et al. [49] demonstrated that the addition of different types of salt ions to the extraction medium can reduce protein solubility through salting-out effects, thereby promoting their precipitation from the solution. In addition, high pH values of the extraction medium can also reduce extrinsic protein contamination [42], leading to less destruction and purification by extrinsic proteins. This step contributes to a high protein yield from OPs.

#### 4.1.2. Selection of Different Solvents for OPs Extraction

Extraction is divided into organic solvent and alkaline solvent methods depending on the properties of the solvents; meanwhile, the conventional approach for OPs extraction uses an organic solvent. A few variables affect extractions, such as type, quantity, and the order of adding reagents. For instance, ether and acetone are typical solvents for degreasing, but only ether-treated samples have fewer lipids (across the whole spectrum) compared with freeze-thawed controls; even in acetone-treated samples, lipid content is high (3.26% phospholipid). A higher optimal degreasing was obtained by controlling the reagent polarity [42]. Nikiforidis et al. [43] achieved higher yields by changing the solubility and intermolecular electrostatic repulsion of the target protein via various methanol-chloroform mixing ratios (95/5, *v*/*v*). In addition, a mixture of methanol, chloroform, and water at a volume ratio of 4:2:1 (*v*/*v*/*v*) facilitates the formation of a biphasic system, which more effectively removes phospholipids, reduces emulsification, and significantly improves the extraction yield of OPs [50]. Moreover, the introduction of an aqueous phase allows hydration interactions with proteins due to its high polarity, helping preserve the native protein structure and avoiding conformational disruption that may occur in pure organic solvent systems, thereby enhancing both extraction efficiency and functional stability of OPs [51]. On this basis, the addition of 1% NaCl to the solvent mixture further increases the OPs content up to 85.3% via salting-out effects and reduces contamination by extrinsic proteins [50]. The aqueous phase plays a key role in modulating solvent polarity and stabilizing the interface, while also providing an ion dissociation environment that enables salt ions to compete with protein surface charges and hydration shells. This promotes protein aggregation and facilitates selective precipitation of OPs, thereby improving both separation efficiency and purity [52]. With the deepening understanding of oleosome structure, Plankensteiner et al. [14] selected high-oil-content rapeseed (40–45%) as the extraction source and precisely regulated the separation behavior of phospholipids, lipids, and proteins. Methanol was first used to disrupt phospholipids on the oleosome surface, followed by hexane to remove oil, and finally ethanol was applied for protein precipitation, yielding OPs with a content as high as 87.1%. This method preserved the hydrophobic domains of OPs and improved their interfacial properties, forming a more robust interfacial membrane. To date, this represents the highest reported OP extraction yield in the literature. Therefore, the choice of plant source is another critical factor affecting the yield and functional properties of OPs [53]. Plant materials with higher oil content, such as rapeseed [54], peanut [55], and hemp seed [56], typically accumulate higher levels of storage proteins and have become a research focus in recent years.

In addition, Wijesundera et al. [46] found that OPs obtained using conventional alkaline extraction often contained lipophilic components, and the yield and purity of OPs could not be effectively guaranteed. To address this, Matsumura, Sirison, Ishi, & Matsumiya [57] adjusted the pH of the extraction system to 5.0 using 0.1 M H_2_SO_4_ and heated the mixture to 55 °C. The increase in temperature promoted the effective separation of the 7S and 11S protein fractions from OPs. Subsequently, 50 mM NaCl solution was added, followed by adjusting the pH of the resulting mixture to 5.5 using 2.0 M NaOH. The extracted protein fractions contained phospholipids such as phosphatidylethanolamine, phosphatidylcholine, and phosphatidylinositol. Due to the highly hydrophobic nature of OPs and the poor staining of phospholipids by Coomassie Brilliant Blue, these components are often invisible on electrophoresis gels. Therefore, OPs obtained through this method show low sensitivity to Coomassie staining and are difficult to detect visually on SDS-PAGE gels. However, clear OP-specific bands (18 kDa and 24 kDa, representing oleosin-associated proteins) were still observed. Compared to other methods, this extraction strategy yielded OPs with superior emulsifying ability and water/oil absorption capacities [57].

Due to the significant hydrophobicity of OPs, their extraction behavior is highly sensitive to aqueous environments, and the extraction efficiency is often lower than that of conventional plant proteins. The strong hydrophobicity and lipophilicity of OPs contribute to their remarkable interfacial stability, yet simultaneously limit their solubility and release under standard alkaline aqueous conditions [58]. Furthermore, OPs tend to form emulsions and stabilize interfacial structures during extraction, which often results in entrapment within lipid droplets, thereby restricting their release [59]. Therefore, the presence of an aqueous phase plays a crucial role in improving OPs extraction efficiency. On one hand, it optimizes the polarity compatibility within the solvent system, facilitating disruption of lipid–protein interfaces and promoting protein release and dispersion [60]. On the other hand, water provides a medium for salt ion dissociation, enhancing competitive hydration against proteins, thus driving selective protein precipitation and improving separation efficiency [61]. However, this aqueous phase factor remains relatively underexplored in current studies. Future efforts in constructing high-efficiency extraction systems should place greater emphasis on the integrative role of water in maintaining system stability, protecting protein structure, and regulating separation behavior.

#### 4.1.3. Separation and Recovery of OPs by Precipitation

Using organic reagents (such as n-hexane) to precipitate proteins or adjusting the isoelectric point of soybean OPs to 5.5 by adding HCl gives the lowest OPs’ solubility, separating the OPs from the solution [62]. Finally, the protein can be dried using freeze-drying, spray-drying or nitrogen-drying. Different drying methods can produce different protein yields and functional properties. Fabre et al. [63] compared the effects of different drying on OPs and found that the spray drying method can more effectively maintain the functional and structural properties of OPs, while the formation of ice crystals during the freeze-drying process will cause structural damage to OPs. Glycerol or Tris can be used as a cryoprotectant to effectively reduce this structural damage and thereby improve its stability. Although research on the effects of drying methods on OPs extraction and functionality remains limited, existing studies and the inherent characteristics of proteins suggest that freeze-drying is currently considered the most conventional and suitable method for preparing OP-based products. This technique effectively removes moisture at low temperatures, thereby minimizing protein denaturation. It is particularly appropriate for applications that require high structural integrity and interfacial activity of proteins, such as emulsion stabilizers, nanoscale delivery systems, and functional foods.

### 4.2. Hybrid Extraction

In addition to these conventional extraction methods, several hybrid extraction strategies have also been employed for isolating OPs, as illustrated in Figure 4. In the hybrid chemical-chemical extraction method, the addition of surfactants significantly enhances protein content. In this process, proteins obtained through chemical extraction further facilitate surfactant extraction, thereby improving extraction efficiency. For example, Sun et al. [7] demonstrated that by soaking seeds in an aqueous medium for protein enrichment, followed by degreasing with n-hexane and acetone, and then treating with a 5 M GuHCl solution, protein content of 75.4% was achieved after dialysis. However, although chemical extraction offers the advantage of high protein yield, the use of strong denaturants such as guanidine hydrochloride (GuHCl) and urea often disrupts the native structure of proteins, alters their functional activity, and may leave behind chemical residues that are difficult to remove. As a result, concerns have been raised regarding the environmental sustainability and food safety of such methods, limiting their direct applicability in functional food systems [64]. Consequently, researchers have increasingly turned to physical processing technologies, such as thermal treatment, in pursuit of improving protein extraction efficiency under milder and more food-compatible conditions.

Heat treatment, as a commonly used physical processing method, can effectively facilitate protein extraction. Zhong et al. [62]. performed dry heat treatment on defatted soybean flour, where an increase in temperature caused protein denaturation and depolymerization, disrupting the secondary and tertiary structures. This process exposed more hydrophilic groups, improving the protein’s nitrogen solubility index. The proteins were then dissolved in an alkaline solution and precipitated in an acidic solution, resulting in a protein content of 76.4%. Although thermal treatment is advantageous due to its operational simplicity and low cost, the irreversible structural changes that proteins undergo during heating limit its application in high-value protein products. For instance, Yang et al. [65] reported that after heating soybean oleosomes at 100 °C for 15 min, OPs experienced irreversible denaturation. The high-temperature treatment led to the exposure of the hydrophobic core of OPs and induced aggregation, resulting in droplet aggregation and increased particle size in emulsions, which ultimately reduced their emulsifying stability. Moreover, such thermal denaturation also suppressed the enzymatic hydrolysis of OPs during digestion. Based on these limitations, the food industry has started to explore milder “non-thermal processing” techniques that aim to enhance extraction efficiency while preserving protein activity. Non-thermal food processing technologies have thus emerged in response to this need.

Mechanical pretreatment, as a physical processing technique, employs strong mechanical forces to facilitate the release of proteins from plant matrices. In the study conducted by Peng et al. [66], the application of twin-screw pressing significantly enhanced the efficiency of the soaking and grinding process. The additional mechanical force facilitated the more effective release of OPs from within the cells into the extraction medium, resulting in a 24.3% increase in OPs content compared to the untreated control group, as shown in Table 2. 

To further improve the extraction efficiency, Qin et al. [73] used a combined method of colloid mill and twin-screw pressing. The addition of a colloid mill further damages the cell wall and can release more proteins, increasing the content of OPs to 60% (*w*/*w*). The results showed that the intensity of mechanical force is a key factor affecting the results of pressing pretreatment. Furthermore, mechanical pretreatment techniques can be combined with chemical extraction techniques. For example, De Chirico, di Bari, Foster & Gray [69] used conventional mechanical stirring by extending the soaking time (16 h) and using 0.1 M NaHCO_3_ with a pH value of 9.5 (solid–liquid ratio 1:7) As a grinding medium, it improves the purity and content of OPs. Romero-Guzmán et al. [74] explored the impact of the combination of salt ion species in the extraction medium and twin-screw technology on the OPs content influence. The results showed that twin-screw pressing destroys the cell structure by applying mechanical force, and combined with salt ion treatment, improves the contact between the solvent and intracellular proteins and promotes the release of OPs. Among them, K^+^ significantly increased the content of OPs (increased by 1.4%) compared with other ion species. Despite the significant improvement in extraction efficiency offered by the aforementioned mechanical methods, their intense shear forces may lead to excessive cell disruption, resulting in heterogeneous protein particle sizes, structural denaturation, and difficulties in subsequent separation. Moreover, such methods impose higher demands on energy consumption and equipment maintenance [75]. Against this backdrop, ultrasound-assisted extraction has attracted increasing attention due to its mild, controllable, and efficient nature [76], and has become a key focus in the field of non-thermal physical extraction technologies in recent years.

Ultrasonic-assisted extraction uses the mechanical vibration caused by ultrasound to increase the contact area between the target protein and the extraction solution. At the same time, the cavitation effect generated by ultrasound destroys the plant cell wall structure, allowing the surrounding solvent to effectively penetrate the cell through the cracks on the cell and release the intracellular protein into the solvent, thereby releasing more protein [77]. Sun et al. [8] used ultrasound-assisted pretreatment to extract OPs and found that the yield of OPs was 16.7% higher after ultrasound pretreatment compared to extraction without ultrasound treatment. Similarly, in the study by Plankensteiner et al. [14], ultrasound pre-treatment resulted in the highest final recovery rate and purity of OPs, reaching 94.0% and 87.1%, respectively.

In summary, mixed extraction methods demonstrate significant potential in the extraction of OPs. Further optimization of these methods is needed, along with exploration of their integration with other technologies. Research indicates that various mixed extraction strategies not only substantially enhance the extraction yield of OPs but also improve their structural and functional properties (Table 2), including particle size, molecular composition, protein secondary, and tertiary structures, as well as interfacial properties. These aspects will be further discussed in subsequent sections.

In summary, hybrid extraction methods can not only significantly improve the yield of OPs but also enhance their structural and functional properties, including particle size, molecular composition, secondary and tertiary structures of proteins, as well as interfacial performance (Table 2). However, current applications of hybrid extraction still face certain bottlenecks. These include process complexity caused by the integration of multiple technologies, increased equipment investment, and challenges in parameter optimization, all of which pose obstacles to industrial feasibility. Moreover, systematic studies on the storage stability, functional persistence, and compatibility of OPs with different food matrices after hybrid extraction remain insufficient. Future research should focus on the efficient integration of mild techniques such as ultrasound with other approaches, the precise synergistic regulation of process parameters, and dynamic analysis of the structure–function relationship of OPs, thereby providing theoretical foundations and technical support for the green, high-value extraction and application of OPs across various scenarios.

In the current processing framework, the extraction of OPs still involves a trade-off among purity, yield, structural preservation, and scalability. Traditional extraction methods (Chemical methods) offer high selectivity and purity but often cause partial denaturation and raise concerns about solvent residues and safety risks during scale-up [78]. Hybrid extraction methods (physical and mechanical treatments) can enhance mass transfer, shorten extraction time, and increase yield, yet excessive stress may disrupt the interfacial membrane and spatial configuration, leading to irreversible structural changes and functional loss [79]. In comparison, hybrid extraction processes that integrate solvent regulation with mild mechanical actions and appropriate system condition adjustments tend to achieve a more stable balance between efficiency and structural integrity. Given that current studies remain largely at the laboratory scale with limited industrial validation, future research should emphasize mild composite extraction strategies developed under application-oriented and sustainable conditions. Within limited-solvent systems, combining moderate energy input with optimized environmental parameters may preserve functional properties while enhancing process scalability, thereby providing practical guidance for industrial implementation.

## 5. Structural Modulation of OPs via Extraction Techniques

### 5.1. Molecular Composition of OPs

The SDS-PAGE results showed that (pH 6.5–11.0), the protein composition of OPs mainly includes intrinsic OPs (oleosin, caleosin, steroleosin) and extrinsic OPs (7S globulin, 11S globulin, 2S albumin, lipoxygenase, trypsin inhibitors, Bd k30, P34, etc.) [74]. For example, in soybean OPs, intrinsic proteins account for 70% of the total protein content, while extrinsic proteins make up about 30% [8]. The protein subunit composition varies among different plant seed sources, and different extraction methods can lead to changes in the protein subunit profile of OPs. As a key factor, pH alters the molecular composition of OPs. Intrinsic proteins in OPs are highly resistant to high pH. Therefore, as the pH increases, the relative content of low-molecular-weight proteins (such as oleosin) becomes more prominent, while the content of extrinsic proteins gradually decreases, significantly enhancing the oxidative stability and storage stability of the oleosome. The probable reason behind this phenomenon is under high pH conditions, the interaction between extrinsic proteins and the surface of the oleosome is weakened, promoting the dissociation of the extrinsic protein from the surface of the oleosome [30,45]. For example, when the extraction pH is increased from 6.8 to 11.0, the extrinsic protein content of soybean and peanut oleosomes gradually decreases, and the oxidative stability increases significantly. It is worth noting that extrinsic proteins outside the oleosome may cause some adverse changes in the physical and chemical properties of the oleosomes, including increased particle size, lowered isoelectric point, and rapid oxidation [72]. Therefore, taking appropriate measures to remove extrinsic proteins is crucial to improve the functional properties of OPs. In addition to adjusting the pH value, the use of surfactants is also an effective way to reduce extrinsic proteins. By adding 9 M urea, Tween 20, 5% sodium dodecyl sulfate, or 5 M guanidine hydrochloride and performing repeated washes during extraction, extrinsic proteins tightly bound to the oleosome surface, especially lipoxygenase, which may promote oxidation, can be effectively removed (dialysis was subsequently performed to eliminate residual reagents and ensure the safety of the obtained oleosomes for food applications) [11]. Meanwhile, during the pre-treatment process of plant grinding and soaking, the application of physical treatments such as ultrasound, colloid mills, and high-pressure homogenization can promote the destruction of cell walls, releasing more extrinsic proteins that interact with the oleosomes, thereby increasing the content of extrinsic proteins in OPs.

### 5.2. Secondary Structure of OPs

The secondary structure of OPs is typically characterized by techniques such as Fourier-Transform Infrared Spectroscopy (FTIR), Circular Dichroism (CD), and Fluorescence Spectroscopy. However, due to the poor water solubility of OPs, FTIR is commonly used to study their secondary structural characteristics. FTIR can effectively analyze the secondary structure of the sample without changing its state and is one of the key methods for structural research on OPs. For example, Qi et al. [80] showed that under acidic conditions, the secondary structure of OPs undergoes some changes. Under alkaline conditions, the α-helix content gradually decreases, while the β-sheet content increases significantly, which indicates that the secondary structure of OPs is more sensitive to alkali treatment than to acid treatment. Similarly, organic reagents can also affect the secondary structure of OPs. Jin et al. [41] reported that OPs extracted with chloroform/methanol exhibited an increase in both α-helix and β-sheet structures compared to OPs obtained with acetone/ether. The results indicate that the choice of organic solvent can affect the separation efficiency of different proteins during the extraction process, especially the chloroform/methanol extraction may enrich more oleosin or steroleosin, leading to an increase in the content of α-helix and β-sheet structures. Furthermore, the addition of salt ions to the extraction medium helps to convert α-helix into β-sheet and β-turns, forming a compact and ordered protein conformation [50]. CD technology is a common method for characterizing protein secondary structure, but due to the poor water solubility of OPs, it is easy to affect the accuracy of data. Therefore, improving solubility is crucial, which could be achieved through ultrasonic pretreatment in the best and most effective way. In the process of increasing ultrasonic power, the cavitation effect of OPs obtained by ultrasonic pretreatment destroys intramolecular hydrogen bonds and enhances the order and stability of the protein, making α-helix and random Curl content decrease, while β-sheet and β-turn content increase [53].

### 5.3. Tertiary Structure of OPs

The tertiary structure of OPs is related to hydrogen bonds, disulfide bonds, electrostatic and hydrophobic interactions, surface hydrophobicity, and free sulfhydryl groups are important indicators of the tertiary structure of OPs. These properties are significantly affected by pH and temperature. Zhao [42] found that P34 and Bd 30K (intrinsic protease) in extrinsic proteins in lipids can pass through disulfide bonds binding to two oleosin of the same molecular weight (18 kDa) leads to the hydrolysis of oleosin, thereby affecting the structure and functional properties of OPs. For this reason, De Chirico [70] found that under specific extraction conditions (pH 11.0 and 60 °C), disulfide bonds in OPs can be broken, removing P34 and Bd 30K, thereby preventing the hydrolysis of oleosin. In addition, physical processing technology will also affect the formation of disulfide bonds of OPs. When the ultrasonic power increases during pretreatment, the high shear force generated by turbulence breaks the hydrogen bonds within the OPs molecule, exposing the internal SH groups to the surface, and promoting disulfide bond formation. The formation of sulfur bonds increases the content of free sulfhydryl groups [50]. In addition, our previous studies have shown that adding chemical reagents significantly impacts the surface hydrophobicity of OPs, and GuHCl can effectively interfere with the non-covalent interactions of proteins when adding OPs during extraction. GuHCl binds to the protein polypeptide chain through the guanidine group, destroying the hydrophobic interaction, causing the protein molecules to present an unfolded loose structure, resulting in the exposure of internal hydrophobic amino acids, thereby increasing the surface hydrophobicity of OPs [8]. However, GuHCl is a non-food-grade surfactant, which limits its application in the food industry. To overcome this limitation, we found that the addition of salt ions to the extraction medium can induce spatial expansion of the OPs structure, thus exposing more hydrophobic groups and increasing the surface hydrophobicity of the obtained OPs [50]. Hydrophobic interactions play a crucial role in protein characteristics, directly determining their structural stability and intermolecular interactions. Therefore, regulating surface hydrophobicity is a key approach to improving the functionality and application performance of proteins [81].

## 6. Improving Physicochemical and Functional Properties Through Extraction Techniques

### 6.1. Particle Size

**Figure 5:**
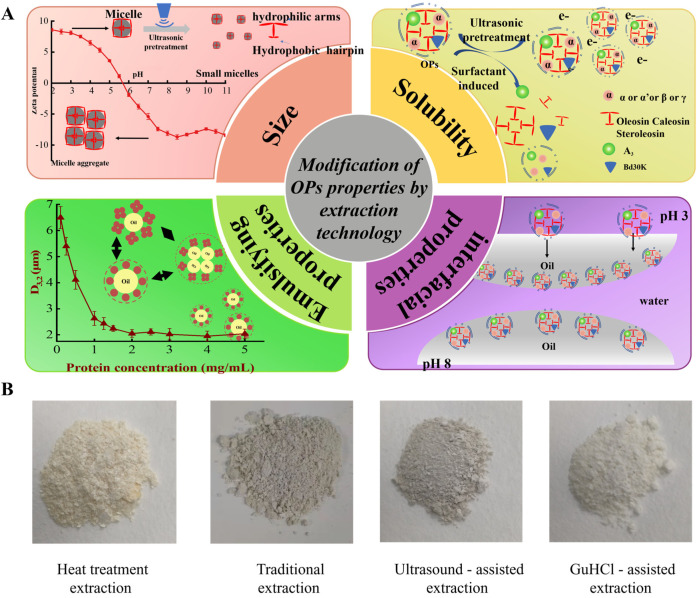
(**A**). Changes in OPs’ physicochemical and functional properties by different extraction techniques; (**B**). Changes in the appearance and structure of OPs by different extraction techniques.

Particle size is an important factor influencing the functional properties of OPs (such as solubility, emulsifying ability, foaming ability, and digestive characteristics). Due to the highly amphiphilic nature of OPs after separation, aggregation occurs in water and most organic solvents due to hydrophobic interactions [82]. pH is typically the main factor affecting the particle size of OPs. Plankensteiner [11] found that pH significantly affected the surface charge distribution of OPs. At pH 3, Oleosin tended to form smaller aggregates, with a size range between 20 and 200 nm, and the main peak is approximately 33 nm. This is closely related to the higher surface charge at lower pH, which increases the electrostatic repulsion between aggregates (Figure 5A). In contrast, at pH 8, TEM and dynamic light scattering observations revealed that the size of OPs aggregates significantly increased, with the main aggregate size ranging from 50 to 400 nm.

The extraction method of OPs has a significant impact on their particle size, and physical processing techniques can notably affect the particle size and size distribution of the extracted OPs. Sun [50] demonstrated that the cavitation and mechanical effects generated by ultrasound-assisted technology during the extraction process caused the OPs particles to expand. As the ultrasound power increased, the particle aggregates gradually dissociated, and the fragmentation of the particles resulted in smaller protein particles. Additionally, Zhong [83] found that, compared to traditional extraction methods, the addition of surfactants (such as SDS) during the extraction process significantly reduced the particle size of OPs, with particle sizes of 354.4 nm and 280.8 nm, respectively. This reduction in particle size is attributed to the hydrophobic interactions of SDS, which induced the dissociation of OPs subunits (α, α′, β, AS, and oleosin), thereby decreasing the particle size.

### 6.2. Solubility

Protein solubility is a key functional property that directly affects emulsifying ability, interfacial properties, and digestibility, thus determining its application in the food industry. However, OPs contain long hydrophobic peptide segments, and their hydrophobic core region is large (accounting for over 50% of their amino acid sequence), resulting in very low water solubility. To address the low solubility of OPs, the surface charge is often regulated to enhance electrostatic repulsion between molecules and reduce aggregation, thereby improving solubility. Adjusting the pH of the solution can increase the net surface charge of OPs, significantly enhancing electrostatic repulsion and inhibiting aggregation. This, in turn, improves solubility. Generally, solubility is lowest when the pH is near the isoelectric point (pI). The isoelectric point of OPs varies depending on the source of the plant seeds. For example, rapeseed OPs have a pI of 7–10 [11], soybean OPs have a pI of 4–6 [80], peanut OPs have a pI of 3–5 [84], and corn germ OPs have a pI of 5–6 [46]. Although solubility increases when moving away from the pI, the effect is limited. For example, soybean OPs have a solubility of only 20.6% at pH 11.0 [50].

In general, the solubility of OPs depends largely on the extraction conditions. Studies have shown that the combination of appropriate physical and chemical treatment methods, such as ultrasonic treatment to destroy hydrophobic interactions, or the addition of salt ions and surfactants to induce spatial structure expansion, can further reduce molecular aggregation. Sun [50] demonstrated that the solubility of OPs obtained via ultrasonic and ultrasound-assisted salt extraction methods was increased by 8.5% and 10.6%, respectively. This increase is attributed to the cavitation effect generated by ultrasound, which disrupted hydrogen bonds between protein molecules, leading to the dissociation of larger protein aggregates into smaller, soluble clusters (as shown in Figure 5A). Additionally, adding salt ions to the ultrasound extraction further enhanced the solubility of OPs. However, even with ultrasound-assisted salt treatment, the maximum solubility of OPs reached only 46.7%, which is comparably low and allows questioning its practical usability. Therefore, other more powerful strategies to increase the solubility of OPs are still under investigation and one efficient method has been proved in which surfactants have been added.

The introduction of surfactants during the extraction process can significantly improve the solubility of OPs. The primary reason for the increase in solubility is the surfactant-induced dissociation of certain protein subunits within OPs (Figure 5A), which alters the molecular weight (Mw) of OPs and enhances electrostatic repulsion. After treatment with reagents such as GuHCl, DTT, and SDS, the solubility of OPs was increased to 68.7% [13], 71.49% [85], and 72.1% [83], respectively. Additionally, the order of adding organic solvents also influences OPs’ solubility. Li [67] studied the effect of different addition sequences of chloroform and methanol on OPs’ solubility, finding that when chloroform was added first followed by methanol, the solubility of OPs was 4.6%, whereas the reverse order led to a solubility of 12.7%. This difference arises from the different order of the delipidation steps, which affects the removal efficiency of neutral lipids and thus leads to differences in the solubility of OPs.

### 6.3. Appearance and Microstructure

The color of foods is an important factor affecting consumer acceptance. Their color characteristics are usually closely related to the purity of the protein, the degree of defatting, and the particle size distribution. For instance, Jin [41] obtained two OPs samples with significantly different structures by changing the addition order of acetone/ether (AE) and chloroform-methanol (CM). OPs-AE presented massive precipitates, while OPs-CM presented a film-like structure. Transmission electron microscopy (TEM) analysis results showed that the surface roughness of OPs-AE was significantly higher than that of OPs-CM, which may be due to the different degreasing effects of the two. Chloroform-methanol can effectively remove most of the phospholipids, making the protein structure more compact and uniform, while acetone/ether has a relatively poor degreasing effect, resulting in a rougher microstructure. Pan [82] further studied the effect of using formic acid to prepare OPs and found that OPs prepared by formic acid formed a slightly turbid colloidal particle dispersion in water with an average particle size of 215.6 nm, with the polydispersity index of 0.238. The morphology of these colloidal particles was observed through TEM and found to be spherical particles with smooth surfaces and uniform particle sizes. Compared with the treatment method using different solvents in the study of Jin [41], OPs prepared by the formic acid method exhibited more uniform structural properties, indicating that the choice of solvent and its interaction with proteins have a significant impact on the structure and color of OPs.

The OPs prepared by heat treatment combined with pH treatment showed a light-yellow block appearance, which was mainly attributed to the lack of organic reagents for defatting during the extraction process and the effect of the heating process on the color of the protein, resulting in the final product showing a unique light-yellow color [62] (Figure 5B). In addition, the OPs pretreated by ultrasound were finer and white powder in appearance compared to the samples without ultrasound treatment (conventional method). Atomic force microscopy showed that OPs without ultrasonic treatment had larger aggregated particles, while after ultrasonic treatment, the particles were significantly smaller, and the structure was more uniform. The results showed that adding physical pretreatment during the extraction process significantly changed the appearance and microstructure of OPs. In addition, adding surfactants (such as GuHCl) during the extraction process of OPs can significantly change their appearance characteristics, and the obtained OPs appeared as tiny and evenly dispersed white particles (Figure 5B). TEM observation results showed that OPs treated with GuHCl exhibited a well-dispersed spherical structure with a particle size of approximately 500 nm, indicating that the use of surfactants improved the appearance and appearance of OPs during the extraction process. The microstructure significantly improved its uniformity and dispersion [8].

### 6.4. Emulsifying Properties of OPs

The emulsifying properties of proteins are a crucial indicator for their practical application in the emulsion system, which is generally expressed by the emulsifying activity index (EAI) and emulsion stabilization index (ESI). In addition, parameters such as droplet size and distribution, flocculation index (FI), and Turbiscan stability (TSI) index can also be used to characterize the emulsification properties of proteins comprehensively. For OPs, the emulsification process is affected by the concentration of the protein suspension and the oil phase content. Generally speaking, higher OPs concentrations help to enhance emulsification activity, but when the protein concentration exceeds the critical concentration, excessive protein may lead to reduced emulsification stability (as shown in Figure 5A). Specifically, when the concentration of soybean OPs increased, the particle size distribution of the emulsion constructed by OPs changed from a bimodal distribution to an unimodal distribution and gradually moved toward smaller particle sizes. However, when the protein concentration exceeded 2.0% (*w*/*w*), the average droplet size remained nearly unchanged while both the TSI and FI values increased [13]. Similarly, high oil phase loading reduces emulsion stability by increasing the collision frequency of oil droplets, which enhances flocculation [82]. Furthermore, at low pH (e.g., pH 3 for rapeseed oleosomes), the higher charge density on the protein particles strengthens electrostatic repulsion between particles, thereby enhancing the stability of the emulsion and reducing the tendency for droplet aggregation. However, when the pH approaches the isoelectric point (e.g., pH 8 for rapeseed oleosomes), the protein’s charge decreases, weakening the electrostatic repulsion and making the oil droplets more prone to flocculation [11].

It is noteworthy that in previous research, it was found that OPs extracted using traditional methods had the lowest EAI and ESI values, with values of 15.4 and 25.3, respectively, at pH 7.4 [8]. After adding surfactants during the extraction process, the EAI and ESI of OPs measured at the same pH (7.4) increased to 24.8 and 31.4, respectively. This difference may be due to differences in solubility and surface hydrophobicity. Proteins with higher surface hydrophobicity and solubility tend to have better emulsification properties [86]. In addition, the extraction method also affects the emulsification properties of OPs. OPs extracted using physical processing pretreatment methods also play an important role in their emulsification characteristics. Sun et al. [50] found that compared with the traditional extraction method (Control-0 W), the EAI and ESI of OPs extracted using ultrasound-assisted extraction increased by 10.3% and 62.7%, respectively. The results showed that ultrasonic treatment not only reduced the particle size but also increased molecular mobility, thereby improving the emulsification ability of OPs.

### 6.5. Interfacial Properties of OPs

At the air–water interface, OPs can form a tight interfacial layer by adsorption, reduce interfacial tension, and thus improve the stability of the bubble film [87]. At the oil–water interface, proteins can also form a protective film by adsorption, effectively preventing the aggregation and fusion of oil droplets and improving the physical stability of the emulsion. Specifically, at low pH (e.g., pH 3 for rapeseed oleosomes), proteins carry a higher charge density, which enhances their uniform distribution at the interface and forms a more compact protective layer, significantly improving emulsion stability (as shown in Figure 5A). However, when pH approaches the protein’s isoelectric point (e.g., pH 8 for rapeseed oleosomes), protein charge diminishes, reducing interfacial adsorption capacity and weakening the tightness and stability of the interfacial layer [14]. Additionally, increasing protein concentration to an optimal level helps form a thicker interfacial membrane, enhancing emulsion stability. However, too high a protein concentration may cause excessive adsorption of proteins on the interface, affecting the uniformity and overall stability of the emulsion [11].

Different extraction methods significantly affect the adsorption behavior of OPs at the air–water and oil–water interfaces and the characteristics of the interfacial film. These interface characteristics are directly related to the stability of the emulsion system. Pan, Jin, & Huang [82] extracted peanut OPs through the anti-solvent precipitation method, forming nanoparticles with an average particle size of 215.6 nm. These nanoparticles formed a tightly packed structure at the oil–water interface, significantly reducing interfacial tension and enhancing emulsion stability. The nanostructure allowed for higher adsorption efficiency at the interface, making this extraction method more favorable for forming stable interfacial films compared to others. Yang et al. [88] further verified the impact of protein behavior at the oil–water interface on the interfacial film properties. They found that OPs form a viscoelastic solid film through in-plane interactions at the oil–water interface. Especially at higher concentrations, this network structure significantly improved the stability of the interfacial film. The results showed that appropriate extraction methods and control of protein concentration can effectively enhance the role of proteins at the interface, thereby improving the stability of the emulsion. Plankensteiner et al. [14] successfully extracted OPs through the sequential extraction method of methanol, hexane, and ethanol (MHE method). The results showed that OPs extracted by the MHE method can form nanoaggregates. These aggregates showed good adsorption capacity at the oil–water interface, reduced the interfacial tension, and formed a stronger interfacial film, which significantly improved the stability of the emulsion. Compared with the traditional method, using methanol, chloroform, and 1% NaCl (Folch method), the effect was more significant. In addition, Li et al. [67] investigated the extraction effect of OPs by changing the sequence combination of degreasing solvents. They found that the combined treatment of Floch solution with diethyl ether and petroleum ether can effectively extract high-purity OPs and retain more phospholipids, so that OPs had better adsorption properties at both the air–water and oil–water interfaces, forming a stable interface structure. This further enhanced the stability of emulsion and foam systems.

Therefore, different extraction methods significantly affect the functional properties of OPs. By choosing an appropriate extraction method, the particle size of OPs can be controlled to make it smaller and better dispersed; its appearance and microstructure can be improved to make the protein particles more uniform; its solubility can be improved to promote its dispersion in the aqueous phase. The reduction in particle size and increase in solubility help to enhance the emulsifying properties of OPs, allowing them to adsorb more effectively at the oil–water and air–water interfaces, reduce interfacial tension, and form a more stable interfacial film. Optimized microstructure and enhanced interfacial properties further prevent the aggregation of oil droplets and air bubbles, ultimately improving the stability of emulsion and foam systems. In conclusion, by adjusting intermolecular interactions, especially through pH control, the functional properties of OPs can be significantly optimized. pH adjustment effectively alters the solubility, particle size, and interfacial adsorption properties of the protein, thereby enhancing emulsifying properties and interfacial stability. Combined with appropriate techniques, these optimization strategies can substantially enhance and improve the overall functional properties of OPs.

## 7. Post-Extraction Modification Techniques for OPs

**Figure 6:**
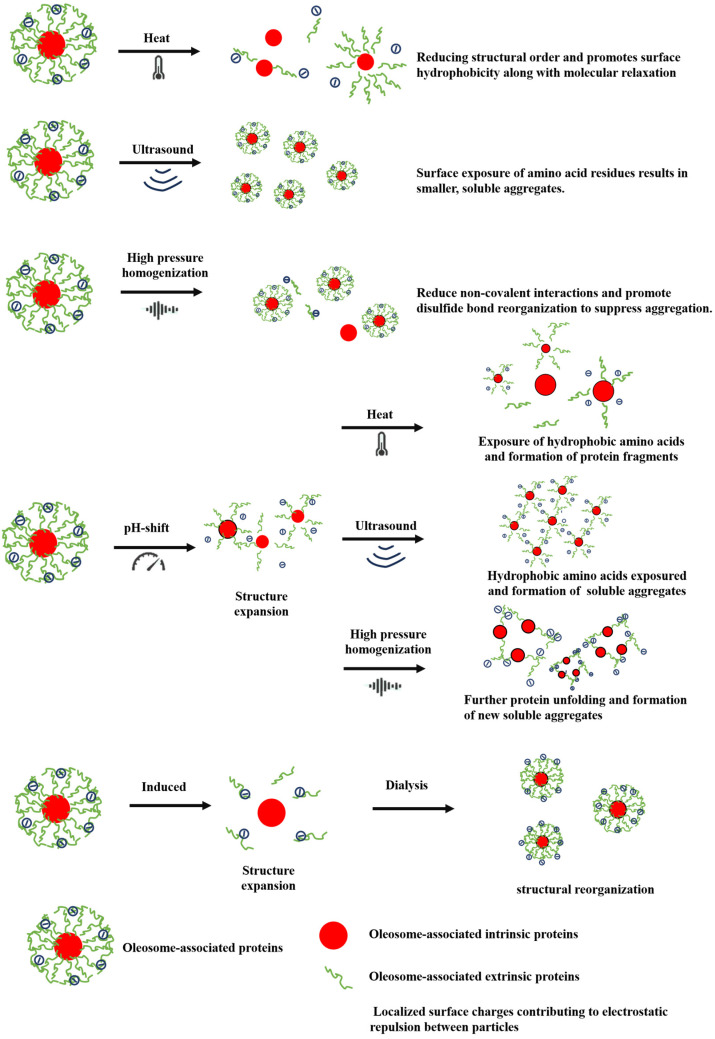
Schematic illustration of the structural changes in OPs induced by different modification techniques.

As discussed earlier, the different extraction methods primarily improve the extraction yield, structure, and functional properties of OPs by adjusting pre-extraction and extraction conditions. However, after the extraction of OPs, further modification techniques can be applied to regulate their molecular structure or chemical groups, thereby improving their functional properties and reducing application limitations [89]. These modifications generally involve various mechanisms, such as reducing particle size, improving solubility, enhancing electrostatic repulsion, and regulating hydrophobic interactions [90]. Protein modification methods are generally classified into two categories: physical and chemical methods, each with its unique mechanism of action (as shown in Figure 6).

### 7.1. Physical Modification

Physical methods are a simple and eco-friendly approach for protein modification, as they do not require chemical reagents or enzymes, avoiding chemical residues. These techniques can alter the structure and functional properties of proteins, thereby enhancing their solubility, emulsifying ability, gelation, and other application properties (as shown in Table 3). Due to the poor solubility of OPs and the presence of hydrophobic amino acids, which tend to aggregate in aqueous solutions, physical methods like heat treatment, ultrasound, or high pressure are commonly employed to improve their dispersibility.

#### 7.1.1. Heat Treatment

Heat treatment disrupts hydrophobic interactions, electrostatic bonds, hydrogen bonds, and disulfide bonds within proteins, leading to structural unfolding. During this process, proteins undergo reversible changes and form intermediate molten states, which improve their functional properties. However, heat treatment may also induce protein aggregation, resulting in a slight decrease in the content of α′, α, and β subunits in OPs molecules [94]. At the same time, heat treatment significantly changes the intermolecular hydrogen bonding caused by water adsorption, resulting in a decrease in α-helical content and an increase in the proportion of random coils. Previous studies have shown that as the random coil content in the secondary structure of OPs increased, the ordered regions in the protein structure gradually decreased, and OPs after heat treatment showed stronger surface hydrophobicity and looser molecular structure [95].

The degree of protein denaturation is closely related to the heating intensity. Appropriate heating can induce structural changes and improve its functional properties, while excessive heating can lead to irreversible denaturation and destroy the stability and functional activity of the protein. Sun et al. [90] found that under pH 2.0 conditions, OPs were heated at 85 °C for 0 to 24 h, and the aggregates gradually transformed into elongated and rigid fibrous structures with increasing heating time. After 6 h of heating, the originally rigid protein fibers became shorter and worm-like, and finally transformed into flexible fibrils. However, high-temperature treatment exceeding 18 h adversely affects the functional properties of OPs, leading to a reduction in solubility or aggravation of protein aggregation. In addition to temperature, the change in pH value of OPs during heat treatment is also another important factor affecting its structural and functional properties. Zhong et al. [94] showed that OPs were treated at pH3.0 and pH11.0 under heating at 60 °C for 30 min, compared with those under pH3.0 conditions. The fluorescence intensity of OPs increased significantly at pH 11.0, and the disappearance of the β-subunit band was observed in SDS-PAGE. The results showed that OPs dissociated under different pH changes and promoted the exposure of the inner surface of the protein.

#### 7.1.2. Ultrasound Treatment

Ultrasonic technology is a green physical processing method that uses high-frequency sound waves to induce cavitation effects in the medium and locally generate high temperature and high pressure, thus changing the molecular structure and functional properties of substances. It is widely used in the field of protein modification [77]. During this process, the particle size and structure of the protein are significantly affected. The cavitation effect generates extremely high temperatures and pressures in local areas through tiny bubbles that form and rapidly collapse in the liquid, thereby destroying the non-uniform interactions between protein molecules. Covalent bonds enhance their surface hydrophobicity and expose more active sites while promoting changes in the secondary and tertiary structures of OPs [18]. Studies showed that after sonication treatment, OPs led to a decrease in α-helix content and an increase in the content of β-sheets and random coils [92]. In addition, ultrasonic treatment can cause some amino acid groups in OPs to be exposed on the surface. The degree of structural change is closely related to the ultrasonic treatment time and power. Low ultrasonic power in a short time can lead to an increase in the particle size of OPs, while long-term high-power ultrasonic treatment can significantly reduce the particle size of OPs. This may be due to the acceleration of protein crushing and dissociation under the action of turbulent force and microcurrent, thereby increasing its hydrophobicity and forming smaller soluble protein aggregates [98]. It is worth noting that no matter how the ultrasonic conditions change, the primary structure of the protein is not affected, which is different from the result of applying ultrasonic treatment when extracting OPs.

In addition, the ultrasonic treatment process can also affect the functional properties of proteins by changing their structure. Ultrasonic treatment can reduce the particle size of OPs, increase their surface hydrophobicity, and thus improve their solubility, which makes the ultrasonically treated OPs useful as an emulsifier for oil-in-water emulsions [96]. Zhong et al. [48] have shown that ultrasound-treated OPs emulsifiers can stabilize emulsion gels, with the gels exhibiting higher viscoelasticity. The density of ultrasound plays a crucial role in the modification process. When the ultrasound power was 200 W, the internal crosslinking of the emulsion gel was more uniform, the pore size smaller, and the stability optimal. Furthermore, OPs emulsions containing hydroxyethyl cellulose exhibited better thermosensitivity. Recently, Honaker, Eijffius, Plankensteiner, Nikiforidis, & Deshpande [13] discovered that ultrasound-treated OPs could form stable chiral nematic liquid crystal structures. These structures show potential as biocompatible alternative materials for bioanalytical detection, and they exhibit sensitivity that could make them suitable for use as high-sensitivity sensors.

#### 7.1.3. High-Pressure Homogenization

High-pressure homogenization is a non-thermal processing technique that uses pressures of 50–300 MPa to force fluids through a narrow gap. Shear, impact, and diffusion forces break non-covalent bonds (such as hydrogen bonds, hydrophobic interactions, and electrostatic forces) within proteins, thereby unfolding the protein structure and exposing polar amino acid residues [73]. This process improves the solubility, emulsifying properties, and surface hydrophobicity of OPs. Gao, Chen, Li, Chi & Teng [84] demonstrated that high-pressure treatment helps unfold the molecular structure of OPs, generates high shear and cavitation effects, and weakens the non-covalent interactions (such as hydrophobic interactions, hydrogen bonds, and electrostatic forces) between proteins, thus reducing aggregation. As the homogenization pressure increases, the surface hydrophobicity of OPs is enhanced, and disulfide bond rearrangement is promoted. Moreover, high-pressure homogenization significantly improves the functional properties of OPs. After treatment at 50, 100, and 150 MPa, OPs exhibited higher solubility, improved emulsifying properties, and more active structural sites. The binding rate of OPs with vitamin B12 was significantly increased, and compared to the control (0 MPa), high-pressure homogenization substantially enhanced the stability and bioavailability of vitamin B12 [95].

### 7.2. Chemical Modification

Chemical modification is a method to regulate the function and stability of food proteins by chemical reactions, adding chemical reagents, or adjusting reaction conditions [96]. This technology can effectively change the three-dimensional structure, hydrophilicity, solubility, and hydrophobic interaction of proteins, thereby improving their biological activity, emulsification, gelation, and other functional properties. In the modification of OPs, commonly used techniques include surfactant-induced modification and regulating the structure and function of proteins by pH shift treatment [8,60], as shown in Table 3.

Surfactants bind to or embed into hydrophobic regions on the surface of proteins, disrupting the secondary and tertiary structures of proteins thereby changing their functional properties. This action may lead to the deconstruction of secondary structures such as α-helices and affect the overall conformation and stability of proteins [60]. In addition, surfactants can alter the surface tension and polarity of the solvent environment, promoting changes in the solubility or aggregation behavior of proteins, thereby affecting their functional properties such as emulsifying capacity, foaming ability, and solubility [8]. Zhong et al. [83] have shown that different surfactants have significantly different impacts on the structure and functionality of OPs. Anionic surfactants like SDS disrupt the hydrophobic interactions of proteins, leading to the unraveling of α-helixes and promoting complete protein denaturation, which enhances emulsifying properties. However, high SDS concentrations may cause protein aggregation, reducing solubility and destabilizing the protein. Similarly, dithiothreitol (DTT), as a reducing agent, disrupts disulfide bonds within proteins, thereby altering their three-dimensional structure. This induces unfolding and refolding of the protein, followed by reassembly, ultimately enhancing the solubility and bioactivity of OPs [85]. GuHCl, a strong denaturant, disrupts non-covalent interactions in proteins, completely denaturing them and leading to the loss of functional conformations, thereby affecting biological functions [8]. In addition, ethanol alters the polarity of the solvent environment, leading to partial protein unfolding or aggregation. Especially at high concentrations, ethanol can promote protein precipitation or aggregation, thereby significantly reducing its solubility and functional properties [97]. These chemical reagents influence protein self-assembly and folding processes, affecting their functional structures. However, the use of surfactants and chemical denaturants is often associated with drawbacks, such as protein aggregation or reduced stability at high concentrations. Consequently, researchers are exploring milder yet effective methods, such as pH shift treatments, to control protein structure and functionality better.

pH shift can significantly alter the structure and functional properties of proteins [93]. By changing the pH, the charge state of surface amino acids in proteins is altered, leading to conformational changes that affect solubility, emulsifying properties, and stability [94]. For OPs, pH variation induces changes in surface charge distribution, reduces the size of protein aggregates, increases surface hydrophobicity and solubility, and modifies intermolecular interactions, causing aggregation, dissociation, or structural unfolding [93]. To achieve more significant protein structural modifications, pH shift is often combined with physical methods such as ultrasound, high-pressure homogenization, and heat treatment. For example, at pH 11.0, OPs combined with ultrasound treatment exhibited more pronounced effects compared to pH shift alone, showing reduced aggregate size, enhanced surface hydrophobicity and solubility, and improved antioxidant activity. This synergistic effect arises from ultrasound-induced local heat and pressure, which facilitates protein unfolding [99]. Notably, the combination of pH shift with high-pressure homogenization and ultrasound significantly enhances the degree of structural modification and effectively generates stable OPs emulsions [62].

## 8. Conclusions and Future Perspectives

In this review, we discussed the structural and functional characteristics of OPs, the current extraction methods and modification strategies, and their effects on the structure and techno-functional properties of OPs. At present, chemical extraction is still a conventional method for obtaining OPs, in which extraction pH and chemical reagents (types and order of addition) are the main factors affecting the structure and functional properties of OPs extracted by chemical means. However, this method has certain problems in terms of biosafety and the preservation of natural structure. In contrast, hybrid extraction methods, through the additional introduction of physical means (such as ultrasound, heating, and colloid milling) combined with chemical regulation, have shown significant advantages in improving OPs extraction efficiency and functional properties. However, the complexity of the process and challenges in industrial application still exist. Therefore, it is necessary to further explore the potential of hybrid extraction methods that combine traditional extraction techniques with various other technologies. By selecting appropriate technical approaches and strictly controlling extraction conditions, the extraction efficiency of OPs and their techno-functional properties can be further improved.

In addition, regarding the modification of OPs, physical methods such as heat treatment, high pressure, and ultrasound have received extensive attention due to their high efficiency and suitability for industrial-scale applications. However, issues such as energy consumption, cost-effectiveness, and sustainability of these physical methods still require further evaluation, and the effect of a single physical treatment is limited. In contrast, chemical modification methods possess stronger precision and structural modification ability at the molecular level, but in practical applications, food safety and protection of natural structure need to be paid special attention. Therefore, it is necessary to further promote an integrated “extraction–modification” strategy based on multi-technology fusion to optimize the precise regulation of the structure and functional properties of OPs.

In addition, the application scenarios of OPs are gradually expanding. As a plant protein with natural interfacial stability, OPs can not only be used for the development of plant-based dairy alternatives, low-fat emulsions, foamed beverages, and functional delivery systems, but also show good nutritional loading capacity and structural optimization effect in elderly foods. They are expected to become a protein used to construct food structures for specific populations. It is worth mentioning that research on the extraction and application of OPs also provides an important model basis for analyzing the interfacial structure and stabilization mechanism of natural oleosomes. This has important theoretical significance for promoting the study of the interface layer in lipid transport and storage mechanisms within plant cells. In conclusion, the extraction and modification technologies of OPs are developing toward multi-technology integration. The research focus has also shifted from the sole improvement of extraction efficiency to the synergistic regulation of structural stability and functional properties, in order to achieve more precise performance construction and high-value utilization under multiple scenarios. Therefore, future research should focus on the integration and optimization of technologies to promote the widespread application of OPs in food and other related fields.

## Figures and Tables

**Table 1:** Conventional Extraction Methods for OPs.

Solvent	Extraction Conditions	Protein Content	Structural Changes	Functional Property Improvements	References
Organic Reagent Extraction					
Acetone, Ether	*v*/*v*, 1/1	NA	Changes in intermolecular components, alteration in protein secondary structure	Increased denaturation temperature	[40]
Chloroform, Methanol	*v*/*v*, 2/1	NA	Changes in intermolecular components, alteration in protein secondary structure	Increased diffusion rate at air–water interface	[41]
Methanol, Hexane, Ethanol	*v*/*v*/*v*, 2/2/1	87.1%	Molecular weight changes	Significant improvement in interfacial properties	[14]
Methanol, Chloroform	*v*/*v*, 95/5	NA	Changes in molecular weight, surface hydrophobicity	Reduced solubility, weakened intermolecular electrostatic repulsion	[42]
Methanol, Chloroform, Water	*v*/*v*/*v*, 4/2/1	74.6%	Changes in protein secondary structure, molecular weight	Changes in emulsifying properties, particle size, water/oil absorption capacity	[8]
Methanol, Chloroform, and 1% NaCl	*v*/*v*/*v*, 4/2/1	75.3%	Molecular weight changes	Changes in particle size, digestion properties, interfacial properties	[43]
Acetone, Methanol, Chloroform	Samples first mixed with equal volumes of acetone, then with a 2:1 (*v*/*v*) mixture of methanol and chloroform	NA	Changes in protein secondary structure, molecular weight	Emulsifying properties	[44]
Ether	Samples mixed with equal volumes of ether	NA	Molecular weight changes	NA	[45]
**Alkaline Extraction**					
Sodium Hydroxide Solution	Plant seed samples soaked in NaOH solution overnight (40%, *w*/*w*), pH adjusted to 12.0	NA	Molecular weight changes	NA	[46]
Sodium Hydroxide Solution with Na_2_SO_3_	Plant seeds soaked in 2 M NaOH (1:8, *w*/*v*) overnight, pH 12.0, then treated with Na_2_SO_3_, heated at 55 °C, and pH adjusted to 5.5	76.4%	Molecular weight, surface hydrophobicity, disulfide bond content	Changes in solubility, particle size, gel properties, water/oil absorption capacity	[47,48]

**Table 2:** Hybrid Extraction Methods for OPs.

Solvent	Extraction Conditions	Protein Content	Structural Improvements	Functional Property Enhancements	References
Urea, acetone, petroleum ether	Centrifuge to collect the cream layer, wash with 0.8 to 6.4 M urea to remove impurities, and recover OPs by treatment with a cold acetone/petroleum ether mixture at equal volumes.	86%	Changes in protein secondary structure and surface hydrophobicity.	Changes in interfacial properties and assembly behavior.	[67]
Diethyl ether, methanol, chloroform, jasmonic acid	Extract the aqueous phase and interfacial layer with ether, then add 1 mL of chloroform/methanol (2:1, *v*/*v*) and treat with 5 μM jasmonic acid	NA	Changes in protein tertiary structure.	Protein activity modification and reduction in off-flavor production	[68]
n-Hexane, acetone, GuHCl	Centrifuge the mixture of n-hexane and acetone (threefold volume), then dialyze the OPs with 5 M GuHCl.	75.4%	Molecular weight, surface hydrophobicity, and changes in protein secondary structure	Solubility, particle size, and changes in emulsifying properties	[8]
Acetone, diethyl ether, urea	Centrifuge the mixture of cold acetone and ether (equal volume), then dialyze the OPs with 8 M urea	NA	Changes in molecular weight, with extrinsic proteins in OPs completely removed.	Particle size decreased, and intermolecular electrostatic repulsion weakened.	[38]
Grinding medium-pH combined treatment	Grind the seeds using NaHCO_3_ buffer (pH 9.5), and recover OPs by organic solvent precipitation.	8.5%	Changes in molecular weight	Changes in intermolecular electrostatic repulsion and increased solubility	[69]
Soaking-salt combined treatment	Soak plant seeds in salt solutions of different pH values, enrich by grinding, and recover OPs using organic solvent precipitation	9.2%	Surface hydrophobicity was altered.	Changes in intermolecular electrostatic repulsion	[70]
Heat-pH combined treatment	Defatted soybean meal is subjected to dry heat treatment at 70 °C for 2 h, then stirred at pH 8.0 for 1 h followed by centrifugation. The supernatant is adjusted to pH 5.0, heated at 55 °C for 15 min, and then 50 mM NaCl solution (pH 5.5) is added	71.6%	Molecular weight, surface hydrophobicity, and protein secondary structure were altered.	Interfacial properties and solubility were altered.	[71]
Ultrasound-assisted salt hybrid extraction	A 0.1 M NaCl solution is added to the plant seed slurry, followed by ultrasound-assisted extraction. After enrichment, OPs are recovered using an organic solvent precipitation method.	78.3%	Changes in molecular weight	Particle size, digestibility, and interfacial properties were altered.	[50]
Twin-screw-assisted pH hybrid extraction	Plant seeds are soaked under different pH conditions, followed by twin-screw pressing-assisted extraction, and finally OPs are recovered using an organic solvent precipitation method.	3.2%	Molecular weight was altered.	Solubility was altered.	[69]
High pressure homogenization assisted pH combined extraction	Plant seeds are soaked under different pH conditions, followed by high-pressure homogenization-assisted extraction. After enrichment, OPs are recovered using an organic solvent precipitation method	34.2%	Molecular weight was altered.	Intermolecular electrostatic repulsion was altered.	[72]
Colloid mill-twin screw combined extraction	Plant seeds are soaked under different pH conditions, followed by twin-screw extrusion combined with colloid milling for assisted extraction. After enrichment, OPs are recovered using an organic solvent precipitation method	21.2%	Molecular weight was altered.	Intermolecular electrostatic repulsion was altered.	[73]

**Table 3:** Hybrid Modification Methods for OPs.

Modification Method	Structural Alteration	Functional Property Changes	References
**Physical Modification**			
Thermal treatment	Changes in secondary structure and surface hydrophobicity	Improves thermal stability, antioxidant activity, and film-forming properties	[91]
Ultrasonic treatment	Changes in secondary structure and surface hydrophobicity	Improves solubility, emulsification, storage stability, gelation; enhances heat sensitivity and biocompatibility	[10,18,92]
High-pressure homogenization	Alters surface hydrophobicity; weakens non-covalent interactions between proteins and promotes disulfide bond reorganization	Improves solubility and emulsification; enhances stability and bioavailability of vitamin B12 as an encapsulant	[73]
**Chemical Modification**			
pH shifting	Changes in secondary and tertiary structure and surface hydrophobicity	Improves solubility, emulsification, and antioxidant activity	[93,94]
pH-Thermal treatment	Changes in secondary and tertiary structure and surface hydrophobicity	Enhances interfacial properties and emulsification for Pickering emulsions; enables high internal phase emulsions	[91,94]
pH-High pressure homogenization	Changes in secondary and tertiary structure and surface hydrophobicity	Improves stability and bioavailability of vitamin B12 as an encapsulant	[95]
pH-Ultrasonic treatment	Changes in secondary structure and surface hydrophobicity	Improves solubility, loading capacity, stability, and bioavailability of vitamin E and quercetin as an encapsulant	[96]
OPs-GuHCl	Changes in secondary and tertiary structure and surface hydrophobicity	Improves solubility, emulsification, and foaming; enhances solubility and bioavailability of curcumin	[8]
OPs-SDS	Changes in secondary and tertiary structure and surface hydrophobicity	Improves solubility and emulsification; enhances solubility, loading rate, and bioavailability of resveratrol as an encapsulant	[83]
OPs-DTT	Changes in secondary and tertiary structure and surface hydrophobicity	Enhances solubility, loading rate, and bioavailability of resveratrol as an encapsulant	[85]
OPs-Ethanol	Changes in secondary and tertiary structure and surface hydrophobicity	Causes aggregation and reduces solubility	[97]

## Data Availability

No new data were created or analyzed in this study. Data sharing is not applicable to this article.

## References

[1] Aliyari A., di Bari V., Ratcliffe L.P., Borah P.K., Dong Y., Gray D. (2025). Characterising the concentration-dependent behaviour of heat-treated sunflower oleosomes at an air-water interface. Food Hydrocoll..

[2] Karefyllakis D., Van Der Goot A.J., Nikiforidis C. (2019). The behaviour of sunflower oleosomes at the interfaces. Soft Matter..

[3] Shi Z., Li K., Meng Z. (2024). Recent trends in oleosomes: Extraction methods, structural characterization, and novel applications. Trends Food Sci. Technol..

[4] Abbasi S., Scanlon M. (2023). Microemulsion: A novel alternative technique for edible oil extraction a mechanistic viewpoint. Crit. Rev. Food Sci. Nutr..

[5] Abdullah W., Zhang H. (2020). Recent advances in the composition, extraction and food applications of plant-derived oleosomes. Trends Food Sci. Technol..

[6] Yuan R., Liu J., Ukwatta R.H., Xue F., Xiong X., Li C. (2024). Artificial oil bodies: A review on composition, properties, biotechnological applications, and improvement methods. Food Chem. X.

[7] Ahmad S., d’Avanzo N., Mancuso A., Barone A., Cristiano M.C., Carresi C., Mollace V., Celia C., Fresta M., Paolino D. (2024). Skin Tolerability of Oleic Acid Based Nanovesicles Designed for the Improvement of Icariin and Naproxen Percutaneous Permeation. ACS Appl. Bio Mater..

[8] Sun Y., Zhong M., Kang M., Liao Y., Wang Z., Li Y., Qi B. (2023). Novel core-shell nanoparticles: Encapsulation and delivery of curcumin using guanidine hydrochloride-induced oleosome-associated protein self-assembly. LWT.

[9] Sun Y., Zhang S., Xie F., Zhong M., Jiang L., Qi B., Li Y. (2021). Effects of covalent modification with epigallocatechin-3-gallate on oleosin structure and ability to stabilize artificial oil body emulsions. Food Chem..

[10] Honaker L.W., Eijffius A., Plankensteiner L., Nikiforidis C.V., Deshpande S.J.S. (2024). Biosensing with Oleosin-Stabilized Liquid Crystal Droplets. Small.

[11] Plankensteiner L., Hennebelle M., Vincken J., Nikiforidis C. (2024). Insights into the emulsification mechanism of the surfactant-like protein oleosin. J. Colloid Interface Sci..

[12] Maurer S., Waschatko G., Schach D., Zielbauer B.I., Dahl J., Weidner T., Bonn M., Vilgis T.A. (2013). The role of intact oleosin for stabilization and function of oleosomes. J. Phys. Chem. B.

[13] Sun Y., Zhong M., Liao Y., Kang M., Qi B., Li Y. (2023). Pickering emulsions stabilized by reassembled oleosome-associated protein nanoparticles for co-encapsulating hydrophobic nutrients. Food Hydrocoll..

[14] Plankensteiner L., Yang J., Bitter J.H., Vincken J.-P., Hennebelle M., Nikiforidis C.V. (2023). High yield extraction of oleosins, the proteins that plants developed to stabilize oil droplets. Food Hydrocoll..

[15] Acevedo-Fani A., Dave A., Singh H. (2020). Nature-assembled structures for delivery of bioactive compounds and their potential in functional foods. Front. Chem..

[16] Karabulut G., Goksen G., Khaneghah A. (2024). Plant-based protein modification strategies towards challenges. J. Agric. Food Res..

[17] Zhang M., Cai S., Wang O., Zhao L., Zhao L. (2024). A comprehensive review on walnut protein: Extraction, modification, functional properties and its potential applications. J. Agric. Food Res..

[18] Zheng X., Zou B., Zhang J., Cai W., Na X., Du M., Zhu B., Wu C. (2024). Recent advances of ultrasound-assisted technology on aquatic protein processing: Extraction, modification, and freezing/thawing-induced oxidation. Trends Food Sci. Technol..

[19] Zhang S., McClements D.J., Zheng R., Yu X., Sun Z., Xie B., Chen Y., Deng Q. (2025). A promising perspective to boost the utilizability of oil bodies: Moderate regulation and modification of interface. Compr. Rev. Food Sci. Food Saf..

[20] Huang A. (1992). Oil bodies and oleosins in seeds. Annu. Rev. Plant Physiol. Plant Mol. Biol..

[21] Shao Q., Liu X., Su T., Ma C., Wang P. (2019). New insights into the role of seed oil body proteins in metabolism and plant development. Front. Plant Sci..

[22] Qu R.D., Huang A.H. (1990). Oleosin KD 18 on the surface of oil bodies in maize. Genomic and cDNA sequences and the deduced protein structure. J. Biol. Chem..

[23] Fujii T. (2017). Coagulation and rheological behaviors of soy milk colloidal dispersions. Biosci. Biotechnol. Biochem..

[24] Hu Z., Wang X., Zhan G., Liu G., Hua W., Wang H. (2009). Unusually large oilbodies are highly correlated with lower oil content in *Brassica napus*. Plant Cell Rep..

[25] Chen E., Tai S., Peng C.C., Tzen J. (1998). Identification of three novel unique proteins in seed oil bodies of sesame. Plant Cell Physiol..

[26] Tzen J.T.C. (2012). Integral proteins in plant oil bodies. Int. Sch. Res. Not..

[27] Jiang P., Tzen J. (2010). Caleosin serves as the major structural protein as efficient as oleosin on the surface of seed oil bodies. Plant Signal Behav..

[28] Asefy Z., Tanomand A., Hoseinnejhad S., Ceferov Z., Oshaghi E.A., Rashidi M. (2021). Unsaturated fatty acids as a co-therapeutic agents in cancer treatment. Mol. Biol. Rep..

[29] Lin L., Tzen J. (2004). Two distinct steroleosins are present in seed oil bodies. Plant Physiol. Biochem..

[30] Nikiforidis C.V. (2019). Structure and functions of oleosomes (oil bodies). Adv. Colloid Interface Sci..

[31] Zaaboul F., Matabaro E., Raza H., Xin B., Duhoranimana E., Cao C., Liu Y.F. (2018). Validation of a simple extraction method for oil bodies isolated from peanuts. Eur. J. Lipid Sci. Technol..

[32] Katavic V., Agrawal G.K., Hajduch M., Harris S., Thelen J. (2006). Protein and lipid composition analysis of oil bodies from two Brassica napus cultivars. Proteomics.

[33] Ishii T., Matsumiya K., Nambu Y., Samoto M., Yanagisawa M., Matsumura Y. (2017). Interfacial and emulsifying properties of crude and purified soybean oil bodies. Food Struct..

[34] Chen Y., Cao Y., Zhao L., Kong X., Hua Y. (2014). Macronutrients and micronutrients of soybean oil bodies extracted at different pH. J. Food Sci..

[35] Lan X., Qiang W., Yang Y., Gao T., Guo J., Du L., Noman M., Li Y., Li J., Li H. (2020). Physicochemical stability of safflower oil body emulsions during food processing. LWT.

[36] Guan M., Lv D., Chen F., Yao F., Lin F. (2025). Recent Advances in the Extraction, Functional Characteristics, and Food Applications of Plant Oleosomes. J. Food Sci..

[37] Yang J., Kornet R., Ntone E., Meijers M.G.J., Van den Hoek I., Sagis L., Venema P., Meinders M., Berton-Carabin C., Nikiforidis C. (2024). Plant protein aggregates induced by extraction and fractionation processes: Impact on techno-functional properties. Food Hydrocoll..

[38] Meza S., Cañizares L., Dannenberg B., Peres B., Rodrigues L., Mardade C., de Leon M., Gaioso C., Egea I., de Oliveira M. (2024). Sustainable rice bran protein: Composition, extraction, quality properties and applications. Trends Food Sci. Technol..

[39] Zhao Y., Tian R., Xu Z., Jiang L., Sui X. (2023). Recent advances in soy protein extraction technology. J. Am. Oil Chem. Soc..

[40] Jin W., Pan Y., Wu Y., Chen C., Xu W., Peng D., Huang Q. (2021). Structural and interfacial characterization of oil bodies extracted from Camellia oleifera under the neutral and alkaline condition. LWT.

[41] Jin W., Yang X., Shang W., Wu Y., Guo C., Huang W., Deng Q., Peng D. (2023). Assembled structure and interfacial properties of oleosome-associated proteins from Camellia oleifera as natural surface-active agents. LWT.

[42] Cao Y., Zhao L., Ying Y., Kong X., Hua Y., Chen Y. (2015). The characterization of soybean oil body integral oleosin isoforms and the effects of alkaline pH on them. Food Chem..

[43] Nikiforidis C., Ampatzidis C., Lalou S., Scholten E., Karapantsios T., Kiosseoglou V. (2013). Purified oleosins at air–water interfaces. Soft Matter..

[44] Ding Y., Ma H., Wang K., Azam S.R., Wang Y., Zhou J., Qu W. (2021). Ultrasound frequency effect on soybean protein: Acoustic field simulation, extraction rate and structure. LWT.

[45] Zhao L., Chen Y., Chen Y., Kong X., Hua Y. (2016). Effects of pH on protein components of extracted oil bodies from diverse plant seeds and endogenous protease-induced oleosin hydrolysis. Food Chem..

[46] Wijesundera C., Boiteau T., Xu X., Shen Z., Watkins P., Logan A. (2013). Stabilization of fish oil-in-water emulsions with oleosin extracted from canola meal. J. Food Sci..

[47] Samoto M., Maebuchi M., Miyazaki C., Kugitani H., Kohno M., Hirotsuka M., Kito M. (2007). Abundant proteins associated with lecithin in soy protein isolate. Food Chem..

[48] Zhong M., Xie F., Zhang S., Sun Y., Qi B., Li Y. (2020). Preparation and digestive characteristics of a novel soybean lipophilic protein-hydroxypropyl methylcellulose-calcium chloride thermosensitive emulsion gel. Food Hydrocoll..

[49] Romero-Guzmán M., Petris V., De Chirico S., di Bari V., Gray D., Boom R., Nikiforidis C. (2020). The effect of monovalent (Na^+^, K^+^) and divalent (Ca^2+^, Mg^2+^) cations on rapeseed oleosome (oil body) extraction and stability at pH 7. Food Chem..

[50] Sun Y., Zhong M., Wu L., Huang Y., Li Y., Qi B. (2022). Effects of ultrasound-assisted salt (NaCl) extraction method on the structural and functional properties of Oleosin. Food Chem..

[51] Pulikkottil Rajan D. (2024). Extraction, isolation, and characterization techniques of structural proteins. Fish Structural Proteins and Its Derivatives: Functionality and Applications.

[52] Shukla D., Schneider C.P., Trout B.L. (2011). Molecular level insight into intra-solvent interaction effects on protein stability and aggregation. Adv. Drug Deliv. Rev..

[53] Şen A., Acevedo-Fani A., Dave A., Ye A., Husny J., Singh H. (2024). Plant oil bodies and their membrane components: New natural materials for food applications. Crit. Rev. Food Sci. Nutr..

[54] Ji L., Feng W., Chen H., Chu Y., Wong A., Zhu Y., Sinatra G., Bramante F., Carrière F., Stocks F. (2025). Rapeseed oleosomes facilitate intestinal lymphatic delivery and oral bioavailability of cannabidiol. Int. J. Pharm..

[55] Rahman M., Farooq S. (2025). Role of peanut oleosomes in the delivery of curcumin embedded in interpenetrating emulsion-filled gels made with whey protein and chitosan. Colloids Surf. A Physicochem. Eng. Asp..

[56] Ma Z., Bitter J.H., Boom R.M., Nikiforidis C.V. (2023). Thermal treatment improves the physical stability of hemp seed oleosomes during storage. LWT.

[57] Matsumura Y., Sirison J., Ishi T., Matsumiya K. (2017). Soybean lipophilic proteins—Origin and functional properties as affected by interaction with storage proteins. Curr. Opin. Colloid Interface Sci..

[58] Yang R., Deng H., Zhao Y., Lin H., Song Y., Zhao L., Miao W., Zheng B. (2025). Interface engineering of plant oil body for an innovative food ingredient: A review. Trends Food Sci. Technol..

[59] Kaur M., Sinha K., Eastmond P.J., Bhunia R.K. (2025). Exploiting lipid droplet metabolic pathway to foster li-pid production: Oleosin in focus. Plant Cell Rep..

[60] Xu C., Wang H., Pan T., Chen H., Liu D., Wang W. (2025). Interfacial Effects Induced by Nanobubbles: Char-acteristics, Consequences, and Applications in Sustainable Food Processing, Commercial Quality, and Human Health. Compr. Rev. Food Sci. Food Saf..

[61] Krolitzki E., Winkler W., Schwaminger S., Berensmeier S. (2025). Bare magnetic iron oxides for binding and selective elution of lactoferrin from acid whey. Colloids Surf. A Physicochem. Eng. Asp..

[62] Zhong M., Sun Y., Qayum A., Liang Q., Rehman A., Gan R., Ma H., Ren X. (2024). Research progress in soybean lipophilic protein (LP): Extraction, structural, techno-functional properties, and high-performance food applications. Trends Food Sci. Technol..

[63] Fabre J., Lacroux E., Cerny M., Vaca-Medina G., Cassen A., Merah O., Valentin R., Mouloungui Z.J.O. (2023). Oil body extraction from oleo-proteaginous seeds and conservation of valuable native compounds. OCL.

[64] Islam F., Saeed F., Afzaal M., Ahmad A., Hussain M., Khalid M.A., Saevan S.A., Khashroum A.O. (2022). Applications of green technologies-based approaches for food safety enhancement: A comprehensive review. Food Sci. Nutr..

[65] Yang X., Zhou L., Wu Y., Ding X., Wang W., Zhang D., Zhao L. (2023). Effect of Heat Treatment on the Digestive Characteristics of Different Soybean Oil Body Emulsions. Foods.

[66] Peng Y., Shan Z., Jia W., Li M., Wen X., Ni Y. (2025). Comparative analysis of twin-screw pressing and blending methods for walnut oleosome extraction: Yield, physical stability, and functionalities. J. Food Eng..

[67] Li Y., Qiao Y., Zhu Y., Shen W., Jin W., Peng D., Huang Q. (2024). Assembly of oleosin during efficient extraction: Altering the sequence of defatting solvents. Food Chem. X.

[68] Kumari S., Memba L.J., Dahuja A., Vinutha T., Saha S., Sachdev A. (2016). Elucidation of the role of oleosin in off-flavour generation in soymeal through supercritical CO_2_ and biotic elicitor treatments. Food Chem..

[69] De Chirico S., di Bari V., Foster T., Gray D. (2018). Enhancing the recovery of oilseed rape seed oil bodies (oleosomes) using bicarbonate-based soaking and grinding media. Food Chem..

[70] De Chirico S., di Bari V., Romero Guzmán M.J., Nikiforidis C., Foster T., Gray D. (2020). Assessment of rapeseed oil body (oleosome) lipolytic activity as an effective predictor of emulsion purity and stability. Food Chem..

[71] Li Y., Zhong M., Xie F., Sun Y., Zhang S., Qi B. (2020). The effect of pH on the stabilization and digestive characteristics of soybean lipophilic protein oil-in-water emulsions with hypromellose. Food Chem..

[72] Qin C., Han M., Fu R., Mei Y., Wen X., Ni Y., Boom R., Nikiforidis C. (2024). Influence of extraction pH and homogenization on soybean oleosome emulsion stability. LWT.

[73] Qin C., Fu R., Mei Y., Wen X., Ni Y., Boom R., Nikiforidis C. (2024). Combining colloid milling and twin screw pressing for oleosome extraction. J. Food Eng..

[74] Romero-Guzmán M., Jung L., Kyriakopoulou K., Boom R., Nikiforidis C. (2020). Efficient single-step rapeseed oleosome extraction using twin-screw press. J. Food Eng..

[75] Kulkarni S.S., Janssen P.H., Dickhoff B.H. (2023). The impact of material chemistry and morphology on attrition behavior of excipients during high shear blending. Powder Technol..

[76] Li Q., Lih T., Clark D., Chen L., Schnaubelt M., Zhang H. (2025). Sonication-assisted protein extraction improves proteomic detection of membrane-bound and DNA-binding proteins from tumor tissues. Nat. Protoc..

[77] Shen L., Pang S., Zhong M., Sun Y., Qayum A., Liu Y., Rashid A., Xu B., Liang Q., Ma H. (2023). A comprehensive review of ultrasonic assisted extraction (UAE) for bioactive components: Principles, advantages, equipment, and combined technologies. Ultrason. Sonochemistry.

[78] Khalid S., Chaudhary K., Aziz H., Amin S., Sipra H.M., Ansar S., Rasheed H., Naeem M., Onyeaka H. (2025). Trends in extracting protein from microalgae Spirulina platensis, using innovative extraction techniques: Mechanisms, potentials, and limitations. Crit. Rev. Food Sci. Nutr..

[79] Timilsena Y.P., Agarwal D., Logan A., Buckow R. (2025). Oleosome extraction: Challenges, innovations, and opportunities for industrial applications. J. Food Eng..

[80] Qi B., Ding J., Wang Z., Li Y., Ma C., Chen F., Sui X., Jiang L. (2017). Deciphering the characteristics of soybean oleosome-associated protein in maintaining the stability of oleosomes as affected by pH. Food Res. Int..

[81] Shao F., Zhang Y., Wan X., Duan Y., Cai M., Hu K., Zhang H. (2025). Regulation in protein hydrophobicity via whey protein-zein self-assembly for improving the techno-functional properties of protein. Food Chem..

[82] Pan Y., Jin W., Huang Q. (2022). Structure, assembly and application of novel peanut oil body protein extracts nanoparticles. Food Chem..

[83] Zhong M., Sun Y., Sun Y., Song H., Zhang S., Qi B., Li Y. (2022). Sodium Dodecyl Sulfate-Dependent Disassembly and Reassembly of Soybean Lipophilic Protein Nanoparticles: An Environmentally Friendly Nanocarrier for Resveratrol. J. Agric. Food Chem..

[84] Gao Y., Chen L., Li L., Chi H., Teng F. (2024). High-pressure homogenization assisted pH-shifting modified soybean lipophilic protein interacting with chitosan hydrochloride: Double emulsion construction, physicochemical properties, stability, and in vitro digestion analysis. Food Hydrocoll..

[85] Zhong M., Sun Y., Song H., Liao Y., Qi B., Li Y. (2023). Dithiothreitol-induced reassembly of soybean lipophilic protein as a carrier for resveratrol: Preparation, structural characterization, and functional properties. Food Chem..

[86] Tian Y., Lv X., Oh D.H., Kassem J.M., Salama M., Fu X. (2024). Emulsifying properties of egg proteins: Influencing factors, modification techniques, and applications. Compr. Rev. Food Sci. Food Saf..

[87] Rehman A., Liang Q., Karim A., Assadpour E., Jafari S.M., Rasheed H., Virk M., Qayyum A., Suleria H., Ren X. (2024). Pickering high internal phase emulsions stabilized by biopolymeric particles: From production to high-performance applications. Food Hydrocoll..

[88] Yang J., Plankensteiner L., de Groot A., Hennebelle M., Sagis L., Nikiforidis C. (2025). The role of oleosins and phosphatidylcholines on the membrane mechanics of oleosomes. J. Colloid Interface Sci..

[89] Rahman M., Byanju B., Lamsal B.P. (2024). Protein, lipid, and chitin fractions from insects: Method of extraction, functional properties, and potential applications. Crit. Rev. Food Sci. Nutr..

[90] Huang J., Zhang M., Mujumdar A.S., Semenov G., Luo Z. (2024). Technological advances in protein extraction, structure improvement and assembly, digestibility and bioavailability of plant-based foods. Crit. Rev. Food Sci. Nutr..

[91] Sun Y., Jia S., Hou Y., Cheng S., Tan M., Zhu B., Wang H. (2025). Novel thyme essential oil-loaded biodegradable emulsion film based on soybean lipophilic proteins for salmon preservation. Food Hydrocoll..

[92] Li Y., Wang D., Zhang S., Zhong M., Zhao C., Xie F., Qi B. (2020). Stability and in vitro simulated release characteristics of ultrasonically modified soybean lipophilic protein emulsion. Food Funct..

[93] Sun Y., Zhang P., Hou Y., Cheng S., Tan M., Zhu B., Wang H. (2024). Enhanced stability and antibacterial efficacy of edible oleogels-in-water high internal phase emulsions prepared from soybean lipophilic protein. Food Hydrocoll..

[94] Zhong M., Sun Y., Sun Y., Fang L., Wang Q., Qi B., Li Y. (2022). Soy lipophilic protein self-assembled by pH-shift combined with heat treatment: Structure, hydrophobic resveratrol encapsulation, emulsification, and digestion. Food Chem..

[95] Gao Y., Gao T., Li L., Chi H., Teng F. (2025). Modification of soybean lipophilic protein based on pH-shifting and high-pressure homogenization: Focus on structure, physicochemical properties and delivery vehicle. Food Chem..

[96] Liu Y., Tan X., Li L., Xie T., Teng F. (2024). Co-encapsulation of vitamin E and quercetin by soybean lipophilic proteins based on pH-shifting and ultrasonication: Focus on interaction mechanisms, structural and physicochemical properties. Food Chem..

[97] Wang S., Miao S., Sun D. (2024). Modifying structural and techno-functional properties of quinoa proteins through extraction techniques and modification methods. Trends Food Sci. Technol..

[98] Zhong M., Sun Y., Song H., Wang S., Qi B., Li X., Li Y. (2023). Ethanol as a switch to induce soybean lipophilic protein self-assembly and resveratrol delivery. Food Chem. X.

[99] Wu D., Wang H., Guo X., Zhang Z., Gao Z., Gao S., Liu Z., Rao S., Meng X. (2023). Insight into the mechanism of enhancing myofibrillar protein gel hardness by ultrasonic treatment combined with insoluble dietary fiber from oat. LWT.

